# Gene transcripts associated with muscle strength: a CHARGE meta-analysis of 7,781 persons

**DOI:** 10.1152/physiolgenomics.00054.2015

**Published:** 2015-10-20

**Authors:** L. C. Pilling, R. Joehanes, T. Kacprowski, M. Peters, R. Jansen, D. Karasik, D. P. Kiel, L. W. Harries, A. Teumer, J. Powell, D. Levy, H. Lin, K. Lunetta, P. Munson, S. Bandinelli, W. Henley, D. Hernandez, A. Singleton, T. Tanaka, G. van Grootheest, A. Hofman, A. G. Uitterlinden, R. Biffar, S. Gläser, G. Homuth, C. Malsch, U. Völker, B. Penninx, J. B. J. van Meurs, L. Ferrucci, T. Kocher, J. Murabito, D. Melzer

**Affiliations:** ^1^Epidemiology and Public Health Group, Institute of Biomedical and Clinical Science, University of Exeter Medical School, Exeter, United Kingdom;; ^2^The National Heart, Lung, and Blood Institute's Framingham Heart Study, Framingham, Massachusetts;; ^3^Population Studies Branch, National Heart, Lung, and Blood Institute, Bethesda, Maryland;; ^4^Department of Functional Genomics, Interfaculty Institute for Genetics and Functional Genomics, University Medicine and Ernst Moritz Arndt University Greifswald, Greifswald, Germany;; ^5^Department of Internal Medicine, Erasmus Medical Centre, Rotterdam, The Netherlands;; ^6^The Netherlands Genomics Initiative-sponsored Netherlands Consortium for Healthy Aging (NGI-NCHA), Leiden/Rotterdam, the Netherlands;; ^7^Department of Psychiatry, VU University Medical Center, Neuroscience Campus Amsterdam, Amsterdam, the Netherlands;; ^8^Hebrew SeniorLife Institute for Aging Research, Boston, Massachusetts;; ^9^RNA mechanisms of complex diseases group, Institute of Biomedical and Clinical Science, University of Exeter Medical School, Exeter, United Kingdom;; ^10^Centre for Neurogenetics and Statistical Genomics, Queensland Brain Institute, University of Queensland, St. Lucia, Brisbane, Australia;; ^11^Section of Computational Biomedicine, Department of Medicine, Boston University School of Medicine, Boston, Massachusetts;; ^12^Department of Biostatistics, Boston University School of Public Health, Boston, Massachusetts;; ^13^The Mathematical and Statistical Computing Laboratory, Center for Information Technology, National Institutes of Health, Bethesda, Maryland;; ^14^Geriatric Unit, Azienda Sanitaria di Firenze, Florence, Italy;; ^15^Institute for Health Services Research, University of Exeter Medical School, Exeter, United Kingdom;; ^16^Laboratory of Neurogenetics, National Institute on Aging, National Institutes of Health, Bethesda, Maryland;; ^17^Clinical Research Branch, National Institute on Aging, Baltimore, Maryland;; ^18^Department of Epidemiology, Erasmus Medical Center Rotterdam, the Netherlands;; ^19^Department of Prosthetic Dentistry, Gerostomatology and Dental Materials, University Medicine Greifswald, Greifswald, Germany;; ^20^Department of Internal Medicine B - Cardiology, Intensive Care, Pulmonary Medicine and Infectious Diseases, University of Greifswald, Greifswald, Germany;; ^21^Unit of Periodontology, Department of Restorative Dentistry, Periodontology and Endodontology, University Medicine Greifswald, Greifswald, Germany; and; ^22^General Internal Medicine Section, Boston University, Boston, Massachusetts

**Keywords:** gene-expression, muscle, strength, blood, human, leukocyte

## Abstract

Lower muscle strength in midlife predicts disability and mortality in later life. Blood-borne factors, including growth differentiation factor 11 (GDF11), have been linked to muscle regeneration in animal models. We aimed to identify gene transcripts associated with muscle strength in adults. Meta-analysis of whole blood gene expression (overall 17,534 unique genes measured by microarray) and hand-grip strength in four independent cohorts (*n* = 7,781, ages: 20–104 yr, weighted mean = 56), adjusted for age, sex, height, weight, and leukocyte subtypes. Separate analyses were performed in subsets (older/younger than 60, men/women). Expression levels of 221 genes were associated with strength after adjustment for cofactors and for multiple statistical testing, including *ALAS2* (rate-limiting enzyme in heme synthesis), *PRF1* (perforin, a cytotoxic protein associated with inflammation), *IGF1R*, and *IGF2BP2* (both insulin like growth factor related). We identified statistical enrichment for hemoglobin biosynthesis, innate immune activation, and the stress response. Ten genes were associated only in younger individuals, four in men only and one in women only. For example, *PIK3R2* (a negative regulator of PI3K/AKT growth pathway) was negatively associated with muscle strength in younger (<60 yr) individuals but not older (≥60 yr). We also show that 115 genes (52%) have not previously been linked to muscle in NCBI PubMed abstracts. This first large-scale transcriptome study of muscle strength in human adults confirmed associations with known pathways and provides new evidence for over half of the genes identified. There may be age- and sex-specific gene expression signatures in blood for muscle strength.

muscle strength correlates with health and physical function, and poor muscle strength in midlife is a strong, independent predictor of health status decline and mortality over 25 yr (40). Sufficient muscle strength in the hands, arms, and legs is needed for everyday functioning; persons with poor strength are at high risk of disability, injury from falls, and other age-related morbidities (15, 25).

Hand-grip is a frequently used summary measure of strength because it correlates well with strength of other key muscles and is relatively easy to measure with high precision; Bohannon et al. (7) reported strong correlations between grip and knee extension strength (Pearson R = 0.77–0.8) in a sample aged 18 to 85 yr, with Samson et al. (43) reporting similar estimates. Muscle strength (including grip strength) is a more important predictor of mortality risk than muscle mass (9, 32), and grip strength (but not muscle mass) was associated with poor physical functioning in older adults (51). The mechanisms underlying the association between lower strength and mortality are not entirely clear, but a recent large-scale multicountry follow-up study (*n* = 142,861) reported that lower grip strength associated most strongly with cardiovascular mortality (28). Current theories emphasize the role of denervation not compensated by adequate reinnervation, mitochondrial dysfunction, cellular senescence, inflammation, changes in microenvironment, and local skeletal changes, among other factors (4, 44).

Studies of heterochronic parabiosis (connecting the blood circulations of young and old mice) found that circulating factors, in particular lower GDF11 (growth and differentiation factor 11) in older mice, explained the lower muscle regenerative capacity in older compared with younger muscle (8, 11, 48). It is well established that circulating factors, such as proinflammatory mediators and hormones (including testosterone), are strong correlates that predict the slope of decline of muscle mass and strength in aging humans (26). These blood-borne factors may function as systemic regulators influencing muscle and may be different from gene expression patterns in muscle itself.

Previous studies of transcriptome associations with muscle strength in humans were conducted predominantly in muscle tissue and are mostly limited by small sample size (30) or focus on candidate genes (34). These studies can be susceptible to false negatives statistical associations due to lack of power or coverage. A transcriptome-wide study of whole blood transcript associations with grip strength conducted by the InCHIANTI Aging Study (mean age 72 yr, 71% ≥72 yr old) found only one gene, *CEBPB* [CCAAT/enhancer-binding protein beta, required for macrophage-mediated muscle repair in a murine model (42)], to be associated with muscle strength in older humans after adjustment for confounders and multiple testing (18). A follow-up study in humans found that *CEBPB* expression increased following exercise-induced muscle damage (6). However, because of the limited sample size of both studies, important transcriptional signals may have been missed.

In the present study we sought to test associations between transcripts expressed in whole blood and hand-grip strength in multiple adult human cohorts. The majority of RNA in whole blood samples is derived from immature erythrocytes and platelets (∼70% from reticulocytes and ∼18% from reticulated platelets); however, these are predominantly globin related and are not actively transcribed by circulating cells (1). The remaining ∼12% of RNA is from circulating white blood cells of all types, driving nonglobin-related gene expression. We have also performed subgroup analyses by age group and sex, to check for heterogeneity in the results. We used a robust meta-analysis framework within four independent cohorts (*n* = 7,781 participants) from the CHARGE (Cohorts for Heart and Aging Research in Genomic Epidemiology) consortium (37) to identify the genes whose levels of expression assessed by blood transcripts were associated with muscle strength.

## METHODS

### Study Sample

Characteristics of the cohorts are presented in Table 1. Complete data for the planned meta-analysis were available for 7,781 participants from four cohorts: the Framingham Heart Study (23) (FHS, *n* = 5,576, ages = 24–90 yr), the InCHIANTI study (13) (*n* = 667, ages = 30–104 yr), the Rotterdam study (20) (RS, *n* = 556, ages = 46–89 yr), and the Study of Health in Pomerania (52) (SHIP, *n* = 982, ages = 20–81 yr) (total *n* = 7,781). The FHS included two related generations of participants (accounted for in the statistical methodology); the FHS *generation 2* (*n* = 2,421, ages = 40–90 yr) and *generation 3* (*n* = 3,155, ages = 24–78 yr) cohorts were included. The Netherlands Study of Depression and Anxiety (35) (NESDA, *n* = 1,989, ages = 18–65 yr) is also reported but was not included in the discovery meta-analysis due to unavailable data on white cell proportions necessary for the meta-analysis protocol. Overall the cohorts were quite similar with respect to sex distribution and sampling methods, differing only by age distribution and lower mean hand-grip strength in the RS. Detailed study design and cohort information has been previously published (21).

**Table 1.** Characteristics of the study cohorts

	Meta-analysis Cohorts					Additional Cohort
Variables	FHS Gen 2*	FHS Gen 3*	InCHIANTI†	RS3†	SHIP†	NESDA
*n*	2,421	3,155	667	556	982	1,989
*n* ≥ 60 yr (%)	1,319 (54)	48 (1)	547 (81)	264 (45)	268 (27)	158 (8)
Sex (male), *n* (%)	1,095 (45)	1,470 (47)	311 (46)	272 (46)	435 (44)	1,328 (67)
Age, yr, mean ± SD	66 ± 8.9	46 ± 8.8	72 ± 15	60 ± 7.9	50 ± 14	42 ± 13
Age, yr, min:max	40: 90	24: 78	30: 104	46: 89	20: 81	18: 65
WBC counts	yes‡	yes	yes	yes	yes	no
Hand-grip strength						
Mean ± SD, kg	31 ± 12	38 ± 12	29 ± 12	25 ± 9.4	38 ± 12	38 ± 12
Min: max, kg	1: 76	5: 84	3: 76	2: 55	11: 73	10: 90
Microarray platform	Affymetrix GeneChip Human Exon 1.0 ST	Affymetrix GeneChip Human Exon 1.0 ST	Illumina HumanHT-12 v3 BeadChip	Illumina HumanHT-12 v4 BeadChip	Illumina HumanHT-12 v3 BeadChip	Affymetrix Human Genome U219 Array

FHS, Framingham Heart Study; RS3, Rotterdam Study 3; SHIP, Study of Health in Pomerania; NESDA, Netherlands Study of Depression and Anxiety; WBC, white blood cell.

* FHS cohorts analyzed together prior to overall meta-analysis.

† Illumina-based cohorts analyzed together prior to overall meta-analysis.

‡ Cell counts imputed in this dataset; see methods. Additional cohort not included in meta-analysis due to data missing from analysis protocol.

Four additional subset analyses were performed. The subsamples available were *1*) older participants ≥60 yr (*n* = 2,402), *2*) younger participants <60 yr (*n* = 5,379), *3*) male participants (*n* = 3,557), *4*) female participants (*n* = 4,224).

### Phenotype

The primary phenotype was hand-grip strength in kilograms (a normally distributed phenotype). In the FHS, hand-grip strength was measured with a Jamar dynamometer with three trials performed in each hand, and the maximum of the six trials for each participant was used in the analysis. In InCHIANTI each participant recorded their maximum grip strength three times in each hand, and the maximum recorded value of the six trials was used. In the Rotterdam Study grip strength of the nondominant hand was measured three times for each participant, and the maximum recorded value was used. In the SHIP cohort participants were asked to press the hand dynamometer firmly for several seconds, once per hand (left and right), and the maximum value was used. Each participant in NESDA was measured twice with a Jamar dynamometer in their dominant hand, with the maximum recorded value used.

### Peripheral Gene Expression Data

Blood samples were drawn from participants and RNA was isolated, reverse-transcribed to cDNA, which was then amplified and hybridized to a microarray individually for each cohort; methods have been described in detail (21). Briefly, the FHS used Affymetrix Human Exon 1.0 ST GeneChips, characterizing the expression of 16,798 unique genes (after exclusion of probe sets with relative log expression mean values <3). The InCHIANTI and SHIP studies used the Illumina HumanHT-12 v3 Expression BeadChip Kit, and the RS used the Illumina HumanHT-12 v4 Expression BeadChip Kit, with 37,348 probes measured on both Illumina platforms (22,911 unique genes; after exclusions of probes expressed above background in <5% of participants this becomes 15,639 unique genes). These four studies in the primary analysis all used PAXgene tubes to isolate and stabilize the RNA, thereby limiting the technical variability between studies. Finally the NESDA cohort utilized the Affymetrix Human Genome U219 Array, with expression information available on 18,212 unique gene identifiers. Quantile normalization and log2 transformation were performed on the gene expression data in each cohort, and both probes and samples were z-transformed. Raw data from gene expression profiling are available online [FHS (*NCBI dbGAP*: phs000363.v7.p8), InCHIANTI (*GEO*: GSE48152), NESDA (NCBI dbGAP: phs000486.v1.p1), RS (*GEO*: GSE33828), and SHIP-TREND (*GEO*: GSE36382)].

Systematically mapping pairs of probes to RefSeq transcripts (one Affymetrix Exon ST and one Illumina HumanHT-12 probe) found 26,746 probe-pairs corresponding to 17,534 unique RefSeq gene symbols. The assignment of a probe to one or more transcripts was performed as described previously (45). For the Illumina arrays, the transcript sequences derived from the 48,803 probe sequences provided in the Illumina annotation file (HumanHT-12_V3_0_R3_11283641_A, version 3.0, 7/1/2010) were mapped against all available mRNA sequences provided in the UCSC genome annotation database (version 06/30/2013) using string matching. Altogether 29,818 probes were successfully mapped to one or more validated mRNAs. Probes that could be mapped neither to a unique mRNA nor to a single annotated RefSeq gene using the UCSC database were flagged accordingly in the annotation file. In total, 27,171 probes (55.7%) were unambiguously associated with a single mRNA or gene. The same method and version of the UCSC database were used for mapping the probes of the Affymetrix GeneChip Human Exon 1.0 ST microarray. For this array, probe sequences were obtained from the annotation file version HuEx-1_0-st-v2.r2 restricting the probes to the main probe types of the core dataset with unique cross hybridization type and combining them at the level of transcript cluster. For this array system, 196,515 probes (86.0%) of 17,876 transcript clusters were unambiguously associated with a single mRNA or gene. Finally, the probes of both array systems were combined based on the same transcripts obtained from the mapping against the UCSC database.

The Human Genome Nomenclature Committee (22) lists 19,060 protein-coding genes (Sept 15, 2014), fewer than the total “unique identifiers” mapped by the two arrays used in the overall meta-analysis; this discrepancy is due to probes on the array mapping to nonprotein-coding transcripts, which we have included under the term “unique genes” or “transcripts” in this manuscript.

### Statistical Analysis

Using the R statistical software (39) and package “lme4” (3) each cohort performed a linear mixed-effects model for each probe in their microarray data, using the probe as the outcome, muscle strength as an independent variable, and with the following covariates included as fixed effects: age, sex, height (cm), weight (kg), cell count estimates (neutrophils, monocytes, basophils and eosinophils), and fasting state (where applicable). By including these factors as covariates in the models our results are independent of interindividual variation in, for example, lymphocyte cell counts. The following covariates were included as random effects: batch (e.g., amplification and/or hybridization), study site (in InCHIANTI), family structure (in FHS), and RNA quality (e.g., RNA integrity number where available). Empirical cell counts were only available in half of the FHS cohort; the rest of the cohort was imputed by partial least-square regression methods.

### Meta-analysis

A sample-size weighted meta-analysis method was used, where we calculated for each probe an overall *P* value and Z-score that together describe the significance of the effect and the direction and magnitude, respectively; this method was chosen over the effect size/standard error method because of the multiple array technologies and technical considerations that differed between the cohorts. The analysis was done using the Meta-Analysis Tool for Genome Wide-Association Scans (METAL) (54), which took the effect size, sample size, and *P* values from the individual cohort results as input (we set the “minor allele,” “major allele,” “minor allele frequency,” and “strand” to the same fixed value for all cohorts and probes, as this package was developed for genome-wide association studies, and these options are not relevant for gene expression data).

For each analysis (the primary analysis, including all individuals, and the four subset analyses) the Illumina-based cohorts (InCHIANTI, RS, SHIP) were meta-analyzed together, as these technologies are very similar, and then we performed a secondary meta-analysis that used the FHS results and the Illumina results as the input; these are the final meta-analysis results reported. This reduced the heterogeneity in the meta-analysis due to array differences between the cohorts.

Before interpretation of the results, probes were excluded if they were expressed in <5% of the sample or if the heterogeneity *P* value calculated by METAL was <0.05. The Benjamini-Hochberg (BH) (5) false discovery rate (FDR) correction was applied to determine the statistically significant probes for each analysis. Validation was defined as a gene with *P* < 0.05 in the NESDA cohort.

### Ontology Enrichment and Network Analysis

The WEB-based GEne SeT AnaLysis Toolkit (WebGestalt) online resource is a method for determining pathway enrichment (56). We conducted a “Gene Ontology” analysis (database version: Nov 11, 2012) and a “Human Phenotype Ontology” analysis (database version: May 20, 2014) that use a systematic approach to phenotype abnormalities to link them into ontologies (53). Default analysis options were selected, including BH multiple testing adjustment, and the list of 17,534 genes included in the meta-analysis we used as the “background.”

A coexpression analysis was performed in the FHS (as the study with the largest sample size) where Spearman correlations were determined between each gene significantly associated with muscle strength, and all other genes (after adjusting the data for the covariates mentioned in the *Statistical Analysis* section). Genes correlated with rho ≥ 0.5 were selected for visualization in Cytoscape (v3.2.1). Ontology enrichment of networks was performed in Cytoscape using the BiNGO plugin (v2.44).

### A Priori Genes Associated with Muscle Function

We selected sets of genes known to influence muscle function for a priori analysis to highlight whether the pathways in muscle tissue are also important in whole blood. Kelch proteins, including KLHL19, KLHL31, KLHL39m, and KLHDC1, are involved in skeletal muscle function and development (8); canonical and noncanonical Wnt signaling plays crucial roles in maintenance and development of skeletal muscle (7); insulin-like growth factors (IGFs) are also known to play roles in muscle growth and homeostasis (9); and finally we studied transforming growth factor-β family members, including myostatin (GDF8) (27) and GDF11. Supplementation of GDF11 in mice was reported to ameliorate the sarcopenia-like phenotype (15). In their 2007 study, Melov et al. (30) identified 586 unique genes expressed in muscle that were associated with endurance exercise training and differed between older and younger men, which we also checked for associations with muscle strength in this analysis.

### Systematic Literature Search for Genes

For each significant gene in the analysis a systematic search of literature was performed by accessing the “publications” list from GeneCards (http://www.genecards.org) (41), which has the advantage of including publications where the gene ID may have changed over time. From this list the title and abstract were downloaded from National Center for Biotechnology Information (NCBI) PubMed (http://www.ncbi.nlm.nih.gov/pubmed). Searches were then made within each publication for the text string “muscle,” and results were counted.

## RESULTS

### Meta-analysis: Genes Associated with Muscle Strength

**Fig. 1.**
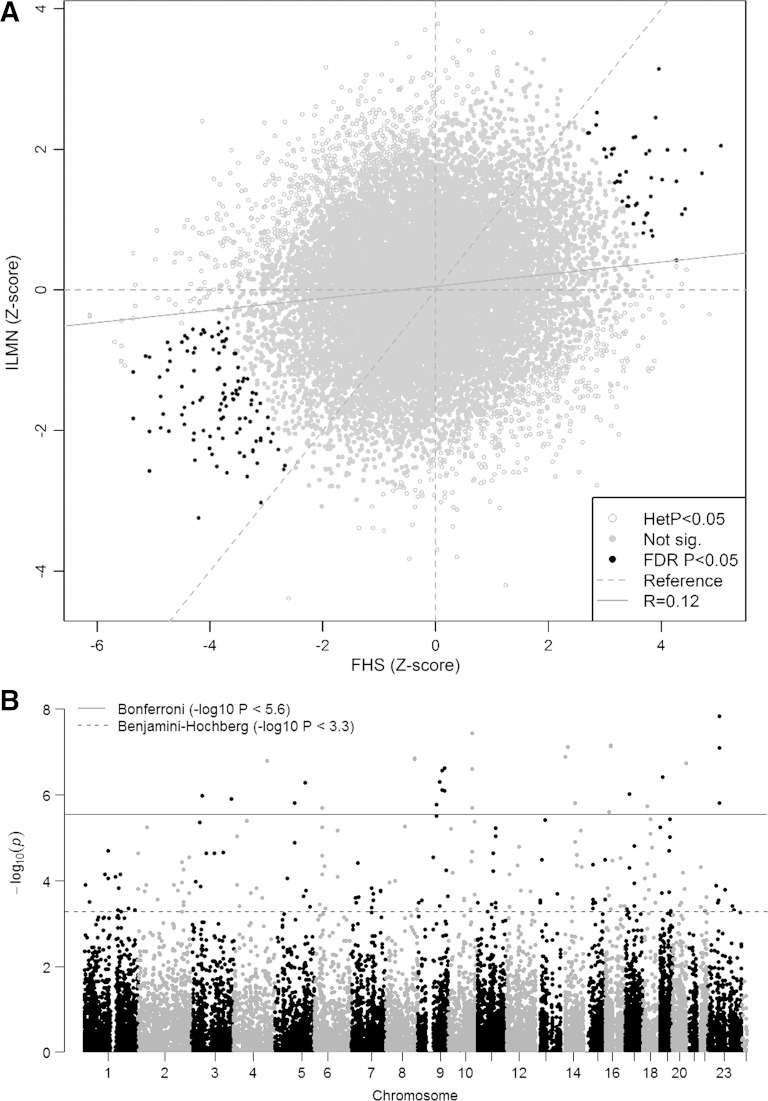
Gene transcripts associated with muscle strength in all participants. *A*: compares the individual meta-analyses performed in the Illumina cohorts and the Framingham Heart Study (FHS) separately. The dark gray points represent gene transcripts significantly associated with muscle strength [false discovery rate (FDR) < 0.05]. The light gray points were not significant in this analysis. The unfilled gray points were excluded because of significant heterogeneity [Cochran's Q-test *P* < 0.05 (54)]. The solid gray line shows the trend across all the genes. *B*: shows the meta-analysis results by Manhattan plot. The dashed line indicates those probes significantly associated with grip strength after Benjamini-Hochberg correction; the solid line shows those significant after Bonferroni correction, for comparison.

Overall, 26,746 probe-pairs (corresponding probes on the Affymetrix and Illumina platforms), mapping to 17,534 unique gene identifiers, were available for the meta-analysis. Including data from all 7,781 participants, 208 unique genes (246 probe-pairs) were associated with muscle strength (FDR < 0.05; Fig. 1, see Supplementary Table S1 for all significant results) after correction for multiple confounders and excluding results with significant heterogeneity (het *P* < 0.05) between cohorts, 133 were negatively associated with grip strength, and 75 were positively associated.^1^

Of the 208 unique genes associated with muscle strength in the meta-analysis all were significant in FHS alone (nominal *P* < 0.05), and 79 (38%) were also “independently” associated with muscle strength in the Illumina meta-analysis (nominally significant, *P* < 0.05). Details of the statistically most significant “top” 20 transcripts are shown in Table 2. The proportion “independently replicated” was greater for the top 30 most significant genes identified in the meta-analysis (21 of 30 = 70%).

**Table 2.** Top 20 unique genes associated with muscle strength in the meta-analysis of all participants, with robust replication in Illumina

*Meta-analysis*			*P* Values				
Z Score	*P* Value	BH *P* Value	FHS	Illumina	Gene	EntrezID	Name
−5.67	1.5 × 10^−8^	2.0 × 10^−4^	3.9 × 10^−7^	9.9 × 10^−3^	*ALAS2*	212	aminolevulinate, delta-, synthase 2
5.37	7.8 × 10^−8^	3.1 × 10^−4^	4.3 × 10^−7^	4.0 × 10^−2^	*HEATR5A*	25938	HEAT repeat containing 5A
−5.28	1.3 × 10^−7^	3.9 × 10^−4^	2.7 × 10^−5^	1.2 × 10^−3^	*PNP*	4860	purine nucleoside phosphorylase
−5.17	2.4 × 10^−7^	4.7 × 10^−4^	1.1 × 10^−6^	5.0 × 10^−2^	*STOM*	2040	stomatin
5.14	2.8 × 10^−7^	4.7 × 10^−4^	7.9 × 10^−6^	1.1 × 10^−2^	*RPS6KA5*	9252	ribosomal protein S6 kinase, 90 kDa, polypeptide 5
−5.09	3.7 × 10^−7^	5.8 × 10^−4^	5.4 × 10^−5^	1.8 × 10^−3^	*MBNL3*	55796	muscle blind-like splicing regulator 3
−5.07	3.9 × 10^−7^	5.8 × 10^−4^	2.3 × 10^−6^	4.4 × 10^−2^	*RAD23A*	5886	RAD23 homolog A (S. cerevisiae)
5.02	5.1 × 10^−7^	6.8 × 10^−4^	7.7 × 10^−5^	1.7 × 10^−3^	*HNRNPA0*	10949	heterogeneous nuclear ribonucleoprotein A0
−4.90	9.5 × 10^−7^	1.1 × 10^−3^	2.0 × 10^−5^	1.5 × 10^−2^	*GID4*	79018	GID complex subunit 4
−4.88	1.1 × 10^−6^	1.1 × 10^−3^	9.3 × 10^−6^	3.4 × 10^−2^	*TFDP1*	7027	transcription factor Dp-1
4.80	1.6 × 10^−6^	1.4 × 10^−3^	9.9 × 10^−6^	4.7 × 10^−2^	*ARRDC3*	57561	arrestin domain containing 3
−4.77	1.7 × 10^−6^	1.5 × 10^−3^	1.8 × 10^−5^	3.3 × 10^−2^	*RIOK3*	8780	RIO kinase 3
−4.71	2.5 × 10^−6^	1.8 × 10^−3^	1.9 × 10^−5^	4.1 × 10^−2^	*NTAN1*	123803	NH_2_-terminal asparagine amidase
4.66	3.1 × 10^−6^	2.2 × 10^−3^	8.1 × 10^−4^	6.0 × 10^−4^	*PDE4D*	5144	phosphodiesterase 4D, cAMP-specific
−4.62	3.8 × 10^−6^	2.4 × 10^−3^	1.1 × 10^−4^	1.2 × 10^−2^	*RGCC*	28984	regulator of cell cycle
4.61	4.1 × 10^−6^	2.5 × 10^−3^	9.6 × 10^−5^	1.4 × 10^−2^	*POLR2B*	5431	polymerase (RNA) II (DNA directed) polypeptide B
−4.59	4.4 × 10^−6^	2.5 × 10^−3^	6.1 × 10^−5^	2.4 × 10^−2^	*EIF1B*	10289	eukaryotic translation initiation factor 1B
4.57	4.8 × 10^−6^	2.7 × 10^−3^	5.4 × 10^−4^	2.0 × 10^−3^	*CIRBP*	1153	cold inducible RNA binding protein
−4.57	4.8 × 10^−6^	2.7 × 10^−3^	6.2 × 10^−5^	2.7 × 10^−2^	*ASGR2*	433	asialoglycoprotein receptor 2
−4.55	5.3 × 10^−6^	2.9 × 10^−3^	3.8 × 10^−5^	4.5 × 10^−2^	*CA1*	759	carbonic anhydrase I

BH, Benjamini-Hochberg. Ordered by meta-analysis *P* value. Showing top 20 results with nominal “replication” (*P* < 0.05) in Illumina. Duplicate gene entries excluded. Full table in Supplementary Table S1.

### Meta-analysis in Subsets of the Participants

The analyses in subsets of the participants identified 13 genes associated with muscle strength at transcriptome-wide significance in the meta-analysis that were not identified in the analysis of all participants together (Table 3, see Supplementary Tables S2–S4 for full results for each subset). We also investigated whether the 208 genes identified in the analysis of all individuals were nominally significant (*P* < 0.05) in the subsets. Of the 208 genes, 153 were nominally significant in the older participants, 198 in the younger, 200 in the men, and 121 in the women (Supplementary Table S1). Supplementary Table S5 includes additional information for all genes included in Tables 2 and 3.

**Table 3.** Thirteen genes associated with muscle strength in age- or sex-specific subset analyses

Subset		Meta-analysis of Subset			*P* Values			
Group	*n*	Z Score	*P* Value	BH *P* Value	FHS	Illumina	Gene	Name
Younger men	5,379	−4.879	1.1 × 10^−6^	9.5 × 10^−3^	2.4 × 10^−6^	1.3 × 10^−1^	*ASAP1*	ArfGAP with SH3 domain, ankyrin repeat and PH domain 1
Younger	5,379	4.495	6.9 × 10^−6^	2.5 × 10^−2^	7.5 × 10^−5^	3.3 × 10^−1^	*PKN2*	protein kinase N2
Younger	5,379	−4.426	9.6 × 10^−6^	2.5 × 10^−2^	2.9 × 10^−5^	6.1 × 10^−1^	*RNF175*	ring finger protein 175
Younger	5,379	−4.407	1.1 × 10^−5^	2.5 × 10^−2^	3.3 × 10^−5^	2.4 × 10^−1^	*TMED5*	transmembrane emp24 protein transport domain containing 5
Younger	5,379	4.235	2.3 × 10^−5^	3.5 × 10^−2^	4.2 × 10^−5^	4.7 × 10^−1^	*ZFYVE27*	zinc finger, FYVE domain containing 27
Younger men	5,379	−4.217	2.5 × 10^−5^	3.5 × 10^−2^	3.4 × 10^−3^	2.5 × 10^−3^	*GID8*	GID complex subunit 8
Younger	5,379	−4	6.3 × 10^−5^	4.2 × 10^−2^	1.7 × 10^−4^	1.2 × 10^−2^	*PIK3R2*	phosphoinositide-3-kinase, regulatory subunit 2
Younger	5,379	−3.961	7.5 × 10^−5^	4.2 × 10^−2^	7.3 × 10^−6^	3.3 × 10^−1^	*PDK4*	pyruvate dehydrogenase kinase, isozyme 4
Younger	5,379	−3.961	7.5 × 10^−5^	4.2 × 10^−2^	2.0 × 10^−6^	6.7 × 10^−1^	*CTNNAL1*	catenin (cadherin-associated protein), alpha-like 1
Younger	5,379	−3.937	8.3 × 10^−5^	4.4 × 10^−2^	7.6 × 10^−5^	1.2 × 10^−1^	*COQ9*	coenzyme Q9
Men	3,557	4.565	5.0 × 10^−6^	1.9 × 10^−2^	1.2 × 10^−5^	1.1 × 10^−1^	*RAC1*	ras-related C3 botulinum toxin substrate 1
Men	3,557	4.041	5.3 × 10^−5^	4.3 × 10^−2^	8.0 × 10^−5^	1.9 × 10^−1^	*NDUFS1*	NADH dehydrogenase (ubiquinone) Fe-S protein 1, 75 kDa ...
Women	4,224	−4.907	9.3 × 10^−7^	2.5 × 10^−2^	8.0 × 10^−4^	1.1 × 10^−4^	*DEFA4*	defensin, alpha 4, corticostatin

Some genes were identified in in multiple groups; for these genes the statistics for the larger group are given; in all cases direction of association is the same. Ordered by subset, then *P* value.

### Significant Genes Only Available on One Array

Due to differences between the array technologies and relative abundance of transcripts, not all the genes were eligible for the meta-analysis. In the analysis on all individuals, 1,123 probes (898 unique identifiers) were present on the Affymetrix Exon array that did not have a corresponding probe on the Illumina array; 21 of these probes were significantly associated with hand-grip strength after BH adjustment for multiple testing (see Table 4 for top 20 probes in the “all individuals” analysis and Supplementary Tables S6–S8 for list of significant probes in each of the FHS analyses with significant results). In the Illumina array, 7,768 probes (6,119 unique gene identifiers) were available that did not map to a gene/transcript in the Affymetrix Exon array (after excluding lowly expressed probes). None of the probes were significantly associated with muscle strength after BH multiple-testing correction.

**Table 4.** Top 20 probes in the FHS analysis that did not map to a corresponding Illumina probe, ordered by P value

Estimate	*P* Value	BH *P* Value	Chr	Start	Gene	Name
−0.0037	1.76 × 10^−6^	1.49 × 10^−3^	1	144989319		
−0.0028	4.89 × 10^−6^	2.88 × 10^−3^	9	37800563	*DCAF10*	DDB1 and CUL4 associated factor 10
0.0031	8.57 × 10^−6^	4.12 × 10^−3^	1	150522766	*ADAMTSL4*	ADAMTS(a disintegrin and metalloproteinase with thrombospondin motifs)-like 4
0.0038	9.29 × 10^−6^	4.15 × 10^−3^	16	89980135		
0.0040	1.95 × 10^−5^	5.97 × 10^−3^	2	162412847	*SLC4A10*	solute carrier family 4, sodium bicarbonate transporter, member 10
0.0029	3.24 × 10^−5^	7.74 × 10^−3^	16	9186734		
0.0031	3.93 × 10^−5^	8.51 × 10^−3^	1	44440179	*ATP6V0B*	ATPase, H+ transporting, lysosomal 21 kDa, V0 subunit b
−0.0027	4.10 × 10^−5^	8.64 × 10^−3^	3	63819562	*THOC7*	THO complex 7 homolog (Drosophila)
0.0032	5.38 × 10^−5^	9.81 × 10^−3^	20	3898284		
0.0019	1.49 × 10^−4^	1.70 × 10^−2^	20	1316212		
0.0018	1.89 × 10^−4^	1.91 × 10^−2^	3	128628719	*ACAD9*/*KIAA1257*	acyl-CoA dehydrogenase family, member 9/KIAA1257
−0.0017	2.21 × 10^−4^	2.08 × 10^−2^	1	8021733	*PARK7*	parkinson protein 7
0.0036	3.18 × 10^−4^	2.69 × 10^−2^	12	57809458		
0.0010	3.20 × 10^−4^	2.70 × 10^−2^	1	153901987	*DENND4B*	DENN/MADD domain containing 4B
−0.0019	3.56 × 10^−4^	2.87 × 10^−2^	3	10157370	*BRK1*	BRICK1, SCAR/WAVE actin-nucleating complex subunit
−0.0028	3.84 × 10^−4^	2.97 × 10^−2^	2	95517671	*TEKT4*	tektin 4
−0.0014	3.95 × 10^−4^	3.03 × 10^−2^	1	247937996	*OR9H1P*	olfactory receptor, family 9, subfamily H, member 1 pseudogene
0.0018	6.01 × 10^−4^	3.89 × 10^−2^	9	124042152	*GSN-AS1*	GSN(gelsolin) antisense RNA 1
0.0028	6.41 × 10^−4^	4.03 × 10^−2^	6	20451305		
−0.0021	6.90 × 10^−4^	4.19 × 10^−2^	12	8024178	*NANOGP1*/*NANOG*	Nanog homeobox pseudogene 1/Nanog homeobox

Blank gene symbols were not annotated to a specific gene. Table continued in Supplementary Table S9. Ordered by *P* value. Not all probes map to gene IDs.

### Ontology Enrichment of Strength-associated Genes

Two analyses were performed to identify pathways using the WebGestalt web resource based on the 208 genes associated with muscle strength in the meta-analysis of all participants.

#### Gene ontology.

Gene ontology analysis found that 10 biological processes were significantly enriched (FDR < 0.05) (Table 5) including “hemoglobin metabolic process” and related processes, “innate immune response” (18 genes) and the stress response (55 genes); 10 molecular functions were enriched (including “protein binding genes”), and 10 cellular component pathways were enriched (including “intracellular membrane-bound organelle”) (See Supplementary Table S9).

**Table 5.** Ten biological processes were enriched in the genes associated with muscle strength in the analysis of all participants

Biological Process	C	O	Raw *P*	Adj. *P*	Top Genes
Hemoglobin metabolic process (GO:0020027)	14	5	3.2 × 10^−7^	0.0004	*ALAS2, AHSP, FECH, EIF2AK2, EPB42*
Hemoglobin biosynthetic process (GO:0042541)	9	3	1.0 × 10^−4^	0.0139	*ALAS2, FECH, EIF2AK2*
Innate immune response (GO:0045087)	539	18	3.8 × 10^−5^	0.0139	*RPS6KA5, IFI27, TLR5, DUSP3, FCGR1B*
Negative regulation of protein metabolic process (GO:0051248)	460	16	6.5 × 10^−5^	0.0139	*IGF2BP2, CIRBP, BANP, GCLC, DUSP3*
Posttranscriptional regulation of gene expression (GO:0010608)	371	14	8.0 × 10^−5^	0.0139	*HNRNPA0, IGF2BP2, EIF1B, CIRBP, ASGR2*
Response to arsenic-containing substance (GO:0046685)	20	4	6.5 × 10^−5^	0.0139	*GCLC, GSTO1, FECH, DDX3X*
Regulation of translational initiation by eIF2a ... (GO:0010998)	2	2	1.0 × 10^−4^	0.0139	*FECH, EIF2AK2*
Regulation of eIF2 alpha phosphorylation by heme (GO:0010999)	2	2	1.0 × 10^−4^	0.0139	*FECH, EIF2AK2*
Response to stress (GO:0006950)	2,952	55	4.7 × 10^−5^	0.0139	*ALAS2, RPS6KA5, RAD23A, HNRNPA0, UBQLN1*
Protoporphyrinogen IX metabolic process (GO:0046501)	11	3	2.0 × 10^−4^	0.0167	*FECH, ALAS2, EIF2AK2*

C, number of reference genes in the category; O, number of genes in the gene set and also in the category; raw P, *P* value from hypergeometric test; Adj. *P*, *P* value adjusted by the multiple test adjustment (Benjamini-Hochberg); Top Genes are the top 5 genes from pathway based on meta-analysis *P* value.

#### Human phenotype ontology.

Human phenotype ontology analysis found 10 phenotypes significantly enriched in the genes, including “anemia due to reduced life span of red cells”, “hemolytic anemia”, and “abnormality of erythrocytes” (see Supplementary Table S10).

Additionally, network analysis in the FHS of all genes correlated (rho ≥ 0.5) with the strength-associated genes (*n* = 425) revealed four clusters with at least 10 genes, all of which had a number of significantly (FDR *P* < 0.05) enriched pathways (Supplementary Table S11): *1*) the largest cluster (*n* = 333 of 425 genes) included “protein ubiquitination,” “erythrocyte homeostasis,” and “cellular metabolic process”; *2*) the second cluster (*n* = 32 genes) was enriched for genes in “regulation of cell communication,” “actin cytoskeleton,” and “ATP metabolic process”; *3*) the third cluster (*n* = 18) had two enriched ontologies only: “cell surface receptor-linked signaling pathway” and “cytolysis”; *4*) the final cluster (*n* = 10) was enriched for terms including “negative regulator or immune system process” and “negative regulation of complement activation.”

### A Priori Genes Associated with Muscle Function

Of 20 *IGF*-related genes tested in this meta-analysis two were significantly associated with muscle strength: *IGF1R* (positively associated) and *IGF2BP2* (negatively associated; meta-analysis FDR = 3.2 × 10^−2^ and FDR = 1.2 × 10^−3^, respectively). Expression of myostation (*MSTN*), follistatin (*FST*), and *GDF11* was not associated with muscle strength (FDR > 0.05). We tested 40 unique Kelch genes in the meta-analysis; none were associated with muscle strength in whole blood (FDR > 0.05). We tested 18 unique Wnt genes (from *WNT1* to *WNT9B*) in the meta-analysis; none were associated with muscle strength in whole blood (FDR > 0.05). All 10 Frizzled genes (*FZD1-10*, receptors for the Wnt pathway) were also available to test; none were associated with muscle strength. Similarly all three Dishevelled genes were available to test (*DVL1-3*, acts directly downstream of the Frizzled receptors), and none were associated with muscle strength. Of 586 genes identified by Melov et al. (30) that were differentially expressed in muscle tissue between old and young men following endurance training, four were associated with muscle strength in this analysis: *ANP32B*, *CIRBP*, *MCM7*, and *MGST1*.

### Most Associated Genes are not Previously Linked to Muscle in the Literature

For each of the 221 genes associated with muscle strength we searched in the published literature cataloged on GeneCards and NCBI Pubmed titles and abstracts, using the search term “muscle”: for 115 of these 221 genes (52%) there were no mentions of muscle (as of Nov 12, 2014, Supplementary Table S12).

### Few Genes Replicate in the NESDA Cohort

NESDA was not included in the meta-analysis due to data limitations: the lack of empirically determined or reliably imputed white cell count data, the use of a different microarray technology (a predecessor to the Exon array used by FHS, much more dissimilar than the v3/v4 Illumina arrays are to one another), and a younger population than the other cohorts included in the meta-analysis (max age = 65 yr, see Table 1).

As noted above, in total 221 unique genes were associated with muscle strength across all the meta-analyses performed. Of 208 genes significantly associated with muscle strength in *analysis 1* (all participants) it was possible to test 144 in the NESDA cohort; seven genes were also associated with muscle strength (*P* < 0.05) in the NESDA cohort (*ACSL6, ALDH5A1, CARHSP1, FGL1, NRG1, PIGB, SIGLEC7*).

### Associations with Knee Strength

Maximum knee and grip strength (both in kg) were measured in 619 participants in the InCHIANTI study and were highly correlated (Pearson R = 0.751) and were significantly associated after adjustment for age, sex, height, and weight (coefficient = 0.193, *P* = 8.2 × 10^−15^) in linear regression models with knee strength as the dependent variable.

## DISCUSSION

In this discovery study we set out to determine whether specific transcript levels in blood are associated with muscle strength in multiple human cohorts including mostly middle-aged volunteers. Previous cross-sectional (and longitudinal) studies have shown that the degree and rate of loss of strength (and muscle mass) is greater in older participants (31). We therefore performed stratified analyses by age and sex to determine whether transcripts or pathways associated with muscle strength in whole blood differ between these groups. We found 208 unique genes to be associated with muscle strength in the analysis of all participants (Table 2 for 20 most robust associations). We identified 13 additional unique genes that were only associated when participants were separated into older/younger or male/female groups (Table 3). In total 221 unique genes were associated with muscle strength in at least one analysis, 52% of which were not previously linked to the term “muscle” in the published literature cataloged on GeneCards and PubMed (as of Nov 12, 2014).

We observe significant associations between muscle strength and expression of *IGF1R* and *IGF2BP2* (positive and negative directions of association with muscle, respectively), growth factors involved in skeletal muscle growth (33, 46); the former is known to enhance cell survival by mediating IGF1 signaling, and the latter modulates *IGF2* translation and has genetic variants associated with Type 2 diabetes (19). Of 586 genes that differ in expression in muscle between old and young men after endurance training (30), four were associated with grip strength in this study: *MGST1* (negative direction), an immune mediator that may protect against oxidative stress (49); *MCM7* (positive direction), which regulates DNA replication during proliferation (12); *CIRBP* (positive direction), which promotes inflammation in response to shock and sepsis (38); and *ANP32B* (negative direction), a cell-cycle progression and antiapoptosis factor (41). These latter results suggest that most blood-based gene expression associated with strength is different from that seen in muscle itself, which is not unexpected given the respective systemic regulatory vs. myofibril maintenance functions involved. Further work should explore whether transcripts that alter in response to exercise show overlaps between circulating cells and muscle. Interestingly, no genes from the Wnt or Kelch pathways [both known to be important for muscle function (14, 29)] were associated with strength in this analysis, nor was *GDF11*, a protein that can reverse age-related muscle dysfunction in mice (48), although as noted we observe associations between strength and expression of two IGF-related genes.

Other genes of note include *CCR6* and *PRF1* [both positively associated with muscle strength and age (17)]: *CCR6* is implicated in B-cell maturation and recruitment (36), and perforin (*PRF1*) is a protein secreted by cytotoxic T-cells that creates pores in membranes to permit apoptosis-inducing granzyme into the target cell (50). These findings are consistent with the notion that inflammation may be associated with muscle repair, maintenance, and turnover, at least in part by interfering with the production and biological activity of IGF-1 (2).

*NANOG* expression was measured in the FHS analysis only and is positively associated with strength in the FHS analysis (Table 4); *NANOG* can reverse aging of some stem cells (16) and, in combination with three other genes (*OCT4*, *SOX2*, and *LIN28*, not significant in this analysis), can induce pluripotency of somatic cells (55). This may suggest that differentiation (of whole blood cells) is inversely correlated with muscle strength, but the mechanisms are unclear.

### Genes Identified in Subset Analyses

Thirteen genes were associated with muscle strength at transcriptome-wide significance in the subset analysis only (Table 3). These were predominantly in the younger (<60 yr) group and included *PIK3R2*, a negative regulator of the PI3K/AKT growth pathway; the negative expression association with strength suggests that there is increased PI3K activity (due to reduced expression of *PIK3R2*) with increasing muscle strength in whole blood. This association is observed in the younger subset (*P* = 6 × 10^−5^) but not in the older subset, even nominally (*P* = 0.92), suggesting differences in growth pathway expression in blood with respect to muscle strength as individuals age. Similarly expression of *PDK4* (inhibits pyruvate dehydrogenase in mitochondria, thereby reducing the conversion of pyruvate into acetyl-CoA) is negatively associated with strength, suggesting increased pyruvate dehydrogenase activity with increased strength in younger individuals only. *PKN2* [associated with height (19) and cell-cycle progression] is positively associated with strength in the younger individuals, underlining the difference in growth pathways in whole blood between younger and older individuals. See Supplementary Table S5 for more details on the other results.

Defensin, alpha 4, Corticostatin (*DEFA4*, negative strength association in the analysis of women only) is a cytotoxic peptide that has antimicrobial activity against Gram-negative bacteria (predominantly) (36). In men, expression of *RAC1* (membrane-associated GTPase involved in signal transduction, including growth signals) and *NDUFS1* (member of mitochondrial complex 1, may form part of the active site) was positively associated with muscle strength; these associations may suggest that on average men have specific energy and growth-related gene expression relating to strength.

No genes were associated (transcriptome-wide) in the older participants only; although the sample size was still reasonably high (2,402 participants), variability in the strength phenotype as individuals age and development of various comorbidities plus chronic inflammation may reduce the power to detect associations. Subset-specific gene expression associations with strength reported here need to be replicated and added to, as we may lack statistical power in this study to detect smaller-effect associations, and the microarrays do not quantify all transcripts or isoforms present.

### Enrichment Analysis

WebGestalt analyses identified statistically significant enrichment for genes in the biological process “hemoglobin metabolic process” and the phenotypic abnormality “hemolytic anemia”, among others. Anemia is a cross-sectional correlate of muscle strength and predicts accelerated muscle strength decline with aging (10), while hemoglobin levels are positively associated with muscle strength and density (10). Circulating reticulocytes (erythrocyte precursors with some residual RNA present) were not adjusted for in this analysis and are likely the source of the associations with genes such as *ALAS2* [strongest meta-analysis association, negative direction, a rate-limiting step in heme biosynthesis (24)].

“Innate immune response” (which includes macrophages) genes were also enriched in the results. *CEBPB*, the gene implicated in the macrophage wound-healing response (42) and significantly associated with muscle strength in the 2012 study by InCHIANTI (18), did not replicate in the other cohorts. This could be due to methodological differences between the previous study and this meta-analysis, as well as differences in age distribution (81% of the InCHIANTI cohort is aged ≥60 yr, compared with 31% in this analysis, which includes the InCHIANTI cohort; Table 1). The implications are unclear given the mouse model evidence of plausible biological mechanism (42) and evidence in humans that exercise-induced muscle damage is associated with *CEBPB* expression changes in whole blood (6). Further work is required in older and frail groups.

Coexpression analysis of all genes correlated with those identified in the meta-analysis to be significantly associated with strength revealed four clusters. Ontology enrichment analysis of these revealed very similar results to those identified using WebGestalt only on the genes significantly associated with strength, emphasizing the association of immune activation and cell signaling pathways to muscle strength in whole blood, in addition to hemoglobin pathways.

### Limitations

There are several potential limitations of this study including its cross-sectional design; it is not possible to determine a causal direction in this study for the associations reported, but the robustly identified markers emerging provide a sound foundation for follow-up studies to address causation. Grip strength is strongly correlated with strength in other key muscle systems (see introduction), but further work will be needed to confirm more specific gene expression associations with strength in other muscle groups. Grip strength can be influenced by nonmuscle strength factors, including functional anomalies in the hands, for example caused by rheumatoid arthritis (47), and work is needed to clarify whether any of our findings reflect these alternative influences.

Another potential limitation is the mixed cell subtype composition of “whole blood”: our analysis approach based on overall expression should have greater power to detect net expression changes in common immune cell types or large changes in expression of highly specific genes but will have less power to detect smaller expression changes within less numerous cell subtypes. The cell subtype origins of the top transcripts reported here now need to be identified. Additionally, the microarray technology used across the participating cohorts was not the same. However, 21 (70%) of the top 30 meta-analysis results were independently replicated between the platforms, which suggests that the top (most strongly associated) results are very robust to cohort and array differences. Also, the current analysis has identified expressed genes statistically associated with muscle strength, but future work will be needed to identify the mechanisms underlying these associations and to determine whether these act on muscle directly or through indirect pathways, perhaps with effects on central command, cerebellar coordination, or neural transmission. Finally, work is needed on whether the identified strength-associated gene expression transcripts are predictive of subsequent changes in strength or functional decline.

### Conclusions

In this first large-scale transcriptome-wide study in human blood, we have identified robust associations between the expression of 221 genes and muscle strength in adults. Several known pathways were confirmed, including growth factor-related genes, the innate immune response, and hemoglobin metabolism. For 115 genes this analysis appears to provide the first published link to muscle. The analysis also suggests that parts of the expression signatures may be specific to subgroups, notably with 10 genes associated with muscle strength only in younger people.

Further work is needed to establish which of the identified genes predict future changes in strength. The findings of genes via expression microarrays may help identify key changes in cell subtypes in blood contributing to strength, through studies of the cellular origins of gene expression signals. Future research should also include longitudinal data to assess whether expression of the identified genes predicts poor muscle strength or functional outcomes.

## DISCLOSURES

No conflicts of interest, financial or otherwise, are declared by the author(s).

## AUTHOR CONTRIBUTIONS

Author contributions: L.C.P., R. Joehanes, M.J.P., D.K., D.P.K., L.W.H., A.T., D.L., H.L., K.L., P.J.M., S.B., W.E.H., D.G.H., A.B.S., T.T., G.v.G., A.H., A.G.U., R.B., S.G., G.H., C.M., U.V., B.W.P., J.B.v.M., L.F., T. Kocher, J.M.M., and D.M. conception and design of research; L.C.P., R. Joehanes, T. Kacprowski, M.J.P., R. Jansen, S.B., D.G.H., A.B.S., and C.M. performed experiments; L.C.P., R. Joehanes, T. Kacprowski, M.J.P., R. Jansen, C.M., and D.M. analyzed data; L.C.P., R. Joehanes, T. Kacprowski, M.J.P., R. Jansen, D.K., D.P.K., L.W.H., A.T., J.E.P., D.L., H.L., K.L., P.J.M., W.E.H., T.T., G.H., B.W.P., J.B.v.M., L.F., T. Kocher, J.M.M., and D.M. interpreted results of experiments; L.C.P. prepared figures; L.C.P. and D.M. drafted manuscript; L.C.P., R. Joehanes, T. Kacprowski, M.J.P., R. Jansen, D.K., D.P.K., L.W.H., A.T., J.E.P., D.L., H.L., K.L., G.H., B.W.P., J.B.v.M., L.F., T. Kocher, J.M.M., and D.M. edited and revised manuscript; L.C.P., R. Joehanes, T. Kacprowski, M.J.P., R. Jansen, D.K., D.P.K., L.W.H., A.T., J.E.P., D.L., H.L., K.L., P.J.M., S.B., W.E.H., D.G.H., A.B.S., T.T., G.v.G., A.H., A.G.U., R.B., S.G., G.H., C.M., U.V., B.W.P., J.B.v.M., L.F., T. Kocher, J.M.M., and D.M. approved final version of manuscript.

## Supplementary Material

Supplementary Table 1

Supplementary Table 2

Supplementary Table 3

Supplementary Table 4

Supplementary Table 5

Supplementary Table 6

Supplementary Table 7

Supplementary Table 8

Supplementary Table 9

Supplementary Table 10

Supplementary Table 11

Supplementary Table 12

## Footnotes

1 The online version of this article contains supplemental material.
